# ADVERSE CHILDHOOD EXPERIENCES AND SLEEP DEPRIVATION IN OLDER ADULTS: THE MEDIATING ROLE OF SOCIAL ISOLATION

**DOI:** 10.1093/geroni/igae098.2509

**Published:** 2024-12-31

**Authors:** Syeda Jesmin, Iftekhar Amin

**Affiliations:** University of North Texas at Dallas, Dallas, Texas, United States; University of North Texas at Dallas, Dallas, Texas, United States

## Abstract

**Background:**

Between 40% and 70% of older adults have chronic sleep issues that can negatively affect their physical and mental health. While research shows that exposure to adverse childhood Experiences (ACEs) can be related to later life sleep quality, it is not clear how social isolation in later life can mediate this relationship. Method: We used the Behavioral Risk Factor Surveillance System (BRFSS) 2022 data. The dependent variable sleep deprivation was measured as sleep less than seven hours per night. Multiple logistic regression models were used to examine the associations between the ACE scores and individual reports of sleep deprivation after controlling for potential confounders. A mediation analysis was estimated for the eight ACE variables, social isolation, and sleep deprivation.

**Results:**

After controlling for gender, race/ethnicity, education, marital status, and self-reported health status, the odds of experiencing sleep deprivation were significantly higher among those who reported four or more ACEs compared to their counterpart who reported three or fewer ACEs (OR = 1.76, 95% CI: 0.171–1.82). Among non-Hispanic Blacks, ACEs were associated with significantly greater odds of reporting sleep deprivation. Social isolation acted as a mediator in the ACE and sleep relationship. An inverse dose-response (P < 0.05) was observed between the sleep duration and experience of ACE.

**Conclusion:**

This study identifies subgroups of older adults who are at greater risk of experiencing sleep deprivation as a result of social isolation and ACEs. Screening for ACEs and improving social participation could help address sleep deprivation in older adults.

